# Salivary Oxidative Stress Markers’ Relation to Oral Diseases in Children and Adolescents

**DOI:** 10.3390/antiox10101540

**Published:** 2021-09-28

**Authors:** Bahareh Nazemi Salman, Shayan Darvish, Ancuta Goriuc, Saeideh Mazloomzadeh, Maryam Hossein Poor Tehrani, Ionut Luchian

**Affiliations:** 1 Department of Pediatric Dentistry, School of Dentistry, Zanjan University of Medical Sciences, Zanjan 4513956184, Iran; drnazemi@zums.ac.ir; 2 Pardis Health Center, Shahid Beheshti University of Medical Sciences, Tehran 1985717443, Iran; 3 Department of Biochemistry, Faculty of Dental Medicine, “Grigore T. Popa” University of Medicine and Pharmacy, 700115 Iasi, Romania; 4 Department of Epidemiology, Zanjan University of Medical Sciences, Zanjan 4513956184, Iran; smazloomzadeh@zums.ac.ir; 5 School of Pharmacy, Tehran University of Medical Sciences, Tehran 1417614411, Iran; m250_tehrani@yahoo.com; 6 Department of Periodontology, Faculty of Dental Medicine, “Grigore T. Popa” University of Medicine and Pharmacy, 700115 Iasi, Romania; ionut.luchian@umfiasi.ro

**Keywords:** oxidative stress, dental caries, saliva, periodontal diseases, child dentistry

## Abstract

Current evidence suggests that salivary markers of oxidative stress are indicative of clinical disease indices such as the papillary bleeding index (PBI) and the caries index (CI). The aim of this study was to assess the relation of oxidative stress markers with oral dental caries and periodontal problems in a pediatric population. In our case-control study, unstimulated whole saliva was collected from individuals aged 3–18 years (*n* = 177); 14 individuals were excluded. Study subjects were divided into those with caries (CI = 2, *n* = 78) and those who were caries-free (*n* = 85). These groups were then divided into another subset consisting of children (mean age 7.3 years, *n* = 121) and adolescents (mean age 16.1 years, *n* = 42). The PBI was determined in all groups. We then assessed salivary levels of oxidative stress markers. Our results showed that, the total antioxidant capacity (TAC) level increased in patients with more gingival bleeding (*p* < 0.05) in the study group aged 3–18 years. In addition, TAC showed a significant decrease in samples with caries when compared to the caries-free group in adolescents (*p* = 0.008). In conclusion, TAC levels may be a marker of both gingival bleeding and dental caries in young adult populations. We hope that in the near future, prophylaxis, control, follow up and even possible therapeutic use of oxidative stress markers in a chairside way will become possible as antioxidants have been shown to be effective against oral diseases.

## 1. Introduction

Dental caries is the most common oral disease found in children and adults. Although preventable, it is still the most common chronic disease in the world and results in pain and tooth loss [1]. Dental caries is five times more prevalent than asthma, which is the second common disease after dental caries in children [2]. Periodontal disease is another frequent oral problem—11% of the global population are suffering from it [3]. Bacteria can cause dental caries and periodontal problems; however, both diseases have a multifactorial etiology, with different biomarkers, that affect saliva [1,4,5]. From these biomarkers, oxidative agents such as reactive oxygen/nitrogen substances (ROS/RNS) take part [6]. They interact and are neutralized by antioxidants such as glutathione, uric acid alpha lipoic acid or vitamin C. [7] Remnants of these free radicals which are not neutralized can cause damage to biochemical molecules such as DNA, lipids, and proteins. This produces different biochemical markers as a byproduct of the damaging process [8]. The imbalance between free radicals and antioxidants is called oxidative stress [9].

**Figure 1:**
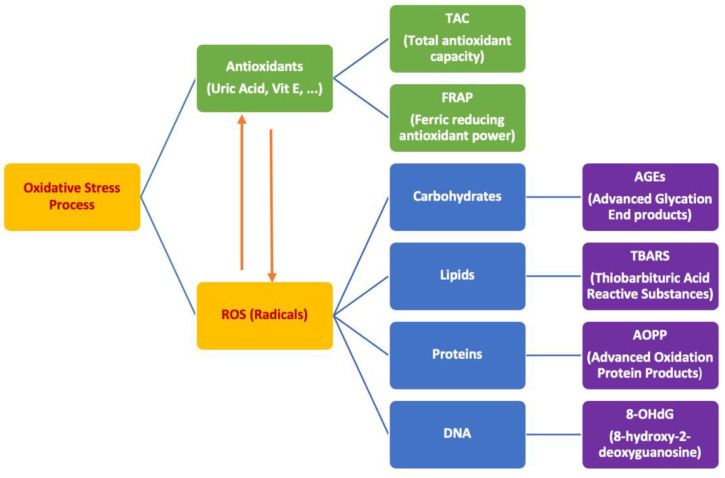
Oxidative stress process and its end product markers.

Previous studies confirm the role of oxidative stress in diabetes, cardiovascular disease, chronic inflammatory diseases, and oral diseases such as dental caries and periodontal problems [10,11,12,13]; however, it is not yet known whether oxidative stress is a cause or a result of the inflammatory response, specifically in oral diseases. Three main possible sources have been introduced for oxidative stress so far: neutrophils, plasma, and bacteria. Among different oxidative stress markers, tiobarbituric acid reactive substances (TBARS) are related to periodontal diseases as a marker for lipid oxidation in saliva [14,15], advanced oxidation protein products (AOPP) are related to dental caries as a marker for protein oxidation [16,17], and total antioxidant capacity (TAC) is a marker for antioxidant agents as it relates to both dental caries and periodontal diseases. Multiple antioxidant agents play a role throughout the body but measuring all of them in saliva is impractical. TAC is a good source to measure overall antioxidant activity [8,18,19], (Figure 1). There is still not enough evidence, however, to use these markers as a way of risk assessment for dental caries or periodontal problems [8].

Lack of research on oxidative stress markers, specifically in a pediatric population, makes it necessary to study them in relation to dental caries and periodontal problems in the valuable fluid of saliva, specifically in children, as early care must be taken to prevent these diseases; thus, this study was designed to assess the possible relation between oxidative stress markers and clinical indices such as PBI and CI in a novel population of children and adolescents with the mean age of 7.3 and 16.1 years, respectively.

## 2. Materials and Methods

### 2.1. Study Group

In this case-control study, 177 healthy children and teenagers between the ages of 3 and 18 were included who had at least one dental cavity infecting the dentin or were caries-free after dental examination (Table 1). The number of cases with at least one tooth with a cavity infecting the dentin that could be identified during routine oral examination was 78 (caries index (CI) = 2). Within the control group, the number of caries-free cases was 85 (CI = 0). All of the borderline cases with possible proximal caries were excluded from the study. Examination and collection of saliva was performed in kindergartens, schools and high schools in Zanjan, Iran. All dental examinations were performed using a single-use sterile oral examination kit and a WHO-type periodontal probe (KerrHawe^®^ SA, Bioggio, Switzerland) by one dental student in their final year of studies under the observation of a periodontist. All kids/teenagers were prohibited from food consumption 2 h prior to saliva collection. Parents were informed with written consent prior to sampling. This procedure was confirmed by the ethical committee of Zanjan University of Medical Sciences (ZUMS.REC.1395.252).

Five milliliters of whole, unstimulated saliva were collected from all children between 9 a.m. and 12 p.m. Saliva samples were stored in Eppendorf tubes and transferred to Tehran University of Medical Sciences, where they were stored at −20 degrees Celsius until the lab test day. PBI examination was performed after saliva sampling to avoid blood contamination using standardized protocols. We assigned PBI = 0 for no bleeding on probing, PBI = 1 for mild bleeding and PBI = 2 for moderate to severe bleeding. The highest score achieved during in the examination period was assigned to each person. Afterwards, the plaque index (PI) was measured on all teeth, using Fuchsin pills and was reported in a percentage.

### 2.2. Lab Procedures

**Figure 2:**
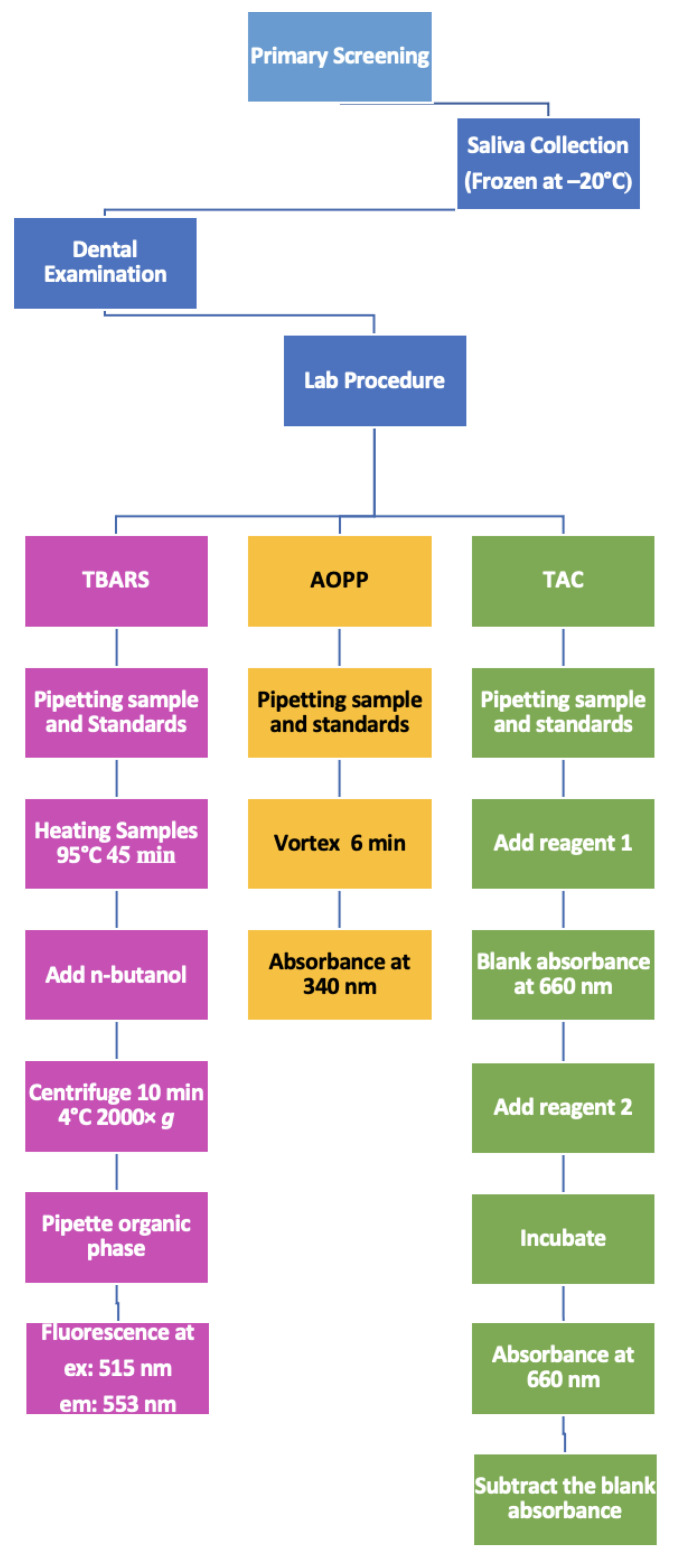
Brief chart for lab procedures measuring TBARS, AOPP, and TAC.

All lab procedures were collected and followed to use the same path to compare the results with the work of Celec et al. and use a standardized method [16]. TBARS, as the marker for lipid oxidation, AOPP, as the marker for protein oxidation and TAC, as the marker for antioxidants, were measured using spectrophotometry and spectrofluorometric methods in this study. A Synergy 4 BioTek multi-mode reader (Winooski, VT, USA) was used to measure the absorbance and fluorescence. All the lab procedures were conducted in Tehran University of Medical Sciences, School of Pharmacy. Briefly, TBARS was measured with n-butanol, along with the heating procedure at excitation: 515 nm and emission at 553 nm, AOPP with glacial acetic acid at 340 nm, and TAC with TROLOX at 660 nm. Lab procedures are shown in Figure 2.

### 2.3. Statistical Analysis

Kolmogorov–Smirnov tests were used first to determine the normal distribution of the data. TAC, TBARS and PI had a normal distribution whereas age and AOPP did not. Independent t-tests and Mann–Whitney U tests were performed for CI, one-way ANOVA and Kruskal–Wallis tests were performed for PBI, chi-squared tests were performed for gender, and post-hoc Tukey’s tests were performed for two-by-two comparisons. SPSS software version 20.0 was used to analyze the data. A *p* value of less than 0.05 was considered a significant relation. In addition to statistical analysis for all samples, samples taken from individuals aged 3–12 and 13–18 was assessed separately as well due to data that suggested different immune system functioning between these ages [20].

## 3. Results

Fourteen samples were excluded from the study due to the exclusion criteria: 1—consumption of any drugs including antibiotics in the past two weeks, 2—food consumption in the two hours prior to sampling, 3—history of any kind of medical or oral diseases except dental caries or periodontal problems. We assessed the relation between CI and oxidative stress markers’ levels in saliva. AOPP, TBARS and TAC level changes were not significant between the caries-free (CI = 0) and caries-infected dentin (extreme caries) (CI = 2) group in overall samples aged between 3 and 18. This made us curious to investigate further; we decided to separate the adolescents (13 to 18, *n* = 42) from children (3 to 12, *n* = 121) due to different immune system functioning between these two age groups. After separating the groups, TAC levels, which represent the total antioxidant capacity of saliva, expressed a significant decrease in adolescents with a caries index of 2 (*p* = 0.008) when compared to the caries-free samples of the same age.

This significant TAC decrease was not expressed in the children’s group aged 3 to 12. AOPP and TBARS did not have significant changes. Plaque index increased significantly in overall samples with caries infecting dentin (*p* = 0.04). Individual results and comparisons are available in Table 2.

**Figure 3:**
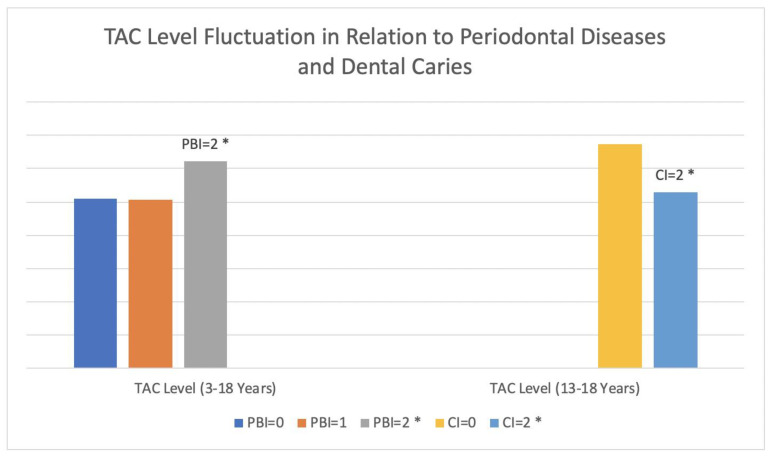
TAC level (µmol/L) fluctuation in relation to periodontal diseases and dental caries. * Indicates *p*-value < 0.05.

Comparing oxidative stress markers and PBI, TAC showed a significant positive correlation with PBI using the ANOVA test (*p* = 0.02). Plaque index also showed a positive significant correlation with PBI (*p* = 0.03). Age of samples had a significant relation with PBI (*p* = 0.0001). No tendency was visible between CI/PBI and gender. Individual results and comparisons for PBI are shown in Table 3. Figure 3 shows the decrease and increase in TAC levels in relation to CI and PBI, respectively.

## 4. Discussion

We found that AOPP as a marker for protein oxidation level was less in the CI = 2 group when compared with caries-free children aged between 3 and 18, though this reduction was not significant. These findings are in opposition to the hypothesis that dental caries can release the marker of protein oxidation in saliva due to the demineralization of dentin proteins. Celecova et al. also reported a significant reduction in AOPP levels. They measured the AOPP level in adults aged between 19 and 83, whereas we measured it in children [21]. A possible explanation for this contrast could be the existence of different mechanisms of inflammation between children and adults. Vahabi et al. showed that during the inflammation process in children, bone/dental degrading materials such as prostaglandins and cytokines will not be secreted; therefore, most of the periodontal diseases will remain at only a gingivitis level in children and will not progress further [20]. Since the inflammation process will not develop completely in children, we did not observe a significant change in the AOPP level. Tothova et al. also did not find any significant relation between CI and AOPP when studying on children between 4 and 18. In their study, the only significant relation between AOPP and CI was observed in adults [16]. Whether significant or not significant, AOPP levels only showed a decrease in these studies, and this makes the hypothesis of AOPP release into saliva during the dental caries process negative. Selmeci et al. explained that AOPP might not even be a proper marker for protein oxidation and may be one of the non-enzymatic oxidative markers’ system [22]. This finding can be a possible explanation for the reduction in AOPP levels in recent studies. More studies are needed, however, to confirm the role of AOPP in oral diseases.

One of the advantages of this study was to divide children and adolescents. As mentioned above, studies show different results when studying children or adults [21,23]. Pyati et al. and Ahmadi-Motamayel et al. showed in their studies that TAC levels increased in the caries active group when studying children and adolescents [24,25]. This made us separate children aged 3 to 12 years and adolescents aged 13 to 18. Again, after separate assessment in children aged 3 to 12 years old, no significant relation was expressed between oxidative stress markers and CI. This might be due to an immature immune system, lack of plasma cell production and an incomplete inflammation process. However, we found that adolescents aged 13 to 18 have a significant decrease in TAC (antioxidant) levels in CI = 2 group, which is counter to the results of the observed studies. A possible explanation for the reduction in TAC levels in teenagers with more severe dental caries could be due to the excess production of free radicals and consumption of antioxidants simultaneously. This is in line with Ahmadi-Motamayel et al.’s work, as they also reported that during the dental caries process, antioxidant levels can decrease [26]. The key point is that during the dental caries procedure, only hard tissue destruction is taking place and the immune system cannot overcome the consumption of antioxidants during the caries process due to lower interaction and involvement. However, periodontal diseases frequently involve the immune system as soft tissue destruction takes place.

We found a significant relation between the TAC marker and the PBI as well. In our study, samples taken from individuals aged 3 to 18 years old with more bleeding upon probing expressed more antioxidant markers (TACs) in their saliva. This is against the finding of multiple studies that expressed lower antioxidant (TAC) levels in the saliva of periodontal patients with more bleeding. Su et al. also showed an increase in antioxidant levels when studying 58 adult periodontal patients and 234 healthy adults. They observed more TAC in the saliva of periodontal patients. First, they attributed this to the possible consumption of antioxidant foods by the periodontal patients, but when they performed the correlation test to remove this effect, they still observed significantly higher TAC levels in periodontal patients [27]. The only explanation that can be given to the TAC increment is the overproduction of antioxidants by the immune system to protect gingival tissues against free radicals. In periodontal diseases, soft and hard tissue destruction takes place simultaneously and the immune system, unlike in dental caries, becomes more involved in the inflammation process, thus increasing the antioxidant agents. 

Zhang et al. had a different result. In their study, TAC levels decreased in the saliva of periodontal patients. They attributed this to the consumption of antioxidants already available in saliva by the immune system to neutralize the free radicals during the inflammation process [28]. More studies are needed to answer the question of whether TAC levels will increase or decrease in severe periodontal conditions. 

Antioxidants have been shown to be a possible effective agent in treating and evaluating oral diseases in recent studies [16]. De Caro et al. showed, in their study, that antioxidants can be used as a thin film to improve the condition of oral diseases in the simple use of cosmetics and nutraceuticals [29]. Zukowski et al. suggested the use of antioxidants in their study as a supplement for people who are exposed to more ROS in their oral cavity [30]. In our study, TAC levels showed both a significant increase and decrease in relation to PBI and CI, respectively. This is in line with the use of antioxidants in oral diseases as suggested by the above studies; thus, these markers can be a proper modifying and controlling factor in determining the oral health status of diseased children.

Celecova et al. did not find any significant relation between CI and TBARS as well but expressed a significant positive relation between TBARS and PBI. In their study, as more bleeding occurs, more periodontal destruction is taking place, and this will release more TBARS in saliva [21]. Celec et al. had the same results. Our results did not emphasize any significant relation between PBI and TBARS in comparison with other studies [26].

It is known that different factors affect oxidative stress markers, free radicals, and antioxidants and future studies should focus on elucidating the bidirectional relationship between them and oral diseases to find the actual source of these markers. The possible sources suggested for production of the free radicals are: 1—the immune system, 2—the actual bacteria present in the inflammation site causing destruction via the free radicals and 3—the exchange between plasma and saliva [8,31]. Alakin et al. studied these markers in chronic periodontitis patients. They compared oxidative stress markers’ levels in saliva, serum and gingival crevicular fluid (GCF). GCF had the highest level of oxidative stress markers in their study. Therefore, the source for the interaction between antioxidants and free radicals can be from localized onsite inflammation [32]. All these factors need to be assessed further to better understand oxidative stress markers.

Plaque index in our study showed a significant increase in more severe bleeding (PBI = 2) and caries-infected dentin (CI = 2) groups. As these plaques are sources of bacteria, it may have affected the production of oxidative stress markers in saliva. Nonetheless, these bacteria and plaque are a part of the inflammation process which contributes to periodontal disease and dental caries originally, and thus can affect the production of oxidative stress markers, but to what extent is still unknown. Interestingly, in adolescents (13–18) the plaque index increased in the CI = 2 group almost significantly (*p* = 0.05) and in the children’s (3–12) group, it had an insignificant increase. Further studies are needed in order to be able to determine the level of effect that plaques can have on this matter.

A possible explanation for different results we found in our study could be external factors that can affect oxidative stress markers and might be a reason for discrepancies. Kamodyova et al. showed that day rhythm can affect oxidative stress markers’ levels. As an example, the AOPP level reached its maximum at 2 p.m. in their study. Another contributing factor in their study was tooth brushing; tooth brushing will increase TAC level as it will wash away the gingival crevicular fluid (GCF) and replace it with fresh saliva full of antioxidants [33]. In our study, we took saliva between 9 a.m. and 12 p.m. to prevent day rhythm difference. We prohibited children to use any food or brush two hours prior to saliva collection; thus, these factors could not have affected our results, but external factors are not limited to these two.

This is one of the first studies that assessed oxidative stress markers and their relation to dental caries and periodontal diseases that differs children and adolescents by considering the effect of puberty and dental system change from deciduous to permanent teeth. All the lab methods were selected from the Celec et al. team who have the most published work on oxidative stress markers regarding oral diseases in order to standardize the methods. Celec et al. mentioned in their recent review article that even the sources of these free radicals are not yet determined and mentions that more interventional studies are needed in the field of oxidative stress [34]. We recommend that antioxidants and their markers can play a key role in relation to oral diseases. Kumar et al. suggested the use of future drugs which rely on understanding the pathway of inflammation and oxidative stress more clearly [35]. We recommend using more interventional studies in relation to oxidative stress markers in all three possible fluids: plasma, saliva and GCF for future studies.

## 5. Conclusions

To conclude, we found a significant positive relation between antioxidant marker (TAC) and PBI, but a negative significant relation was expressed in adolescents aged between 13 and 18 when assessed with dental caries index in this study. TAC level increases in the PBI = 2 group may be associated with the overproduction of antioxidants by the immune system, whereas the TAC level reduction in the adolescent group with severe dental caries (CI = 2) can be attributed to consumption of antioxidant capacities. The incapacity of the immune system to regulate those variations can be influenced by the fluctuations induced by puberty and a change in the dental system.

Our study opens promising perspectives regarding the important role of a potential therapeutic use of oxidative stress markers in a chairside way, as antioxidants have shown to be effective against oral diseases.

## Figures and Tables

**Table 1:** Characteristic information of the study.

	Males	Females	*p*-Value
CI = 0	41 (48.8%)	43 (51.2%)	0.75
CI = 2	40 (51.3%)	38 (48.7%)	
PBI = 0	28 (49.1%)	29 (50.9%)	0.32
PBI = 1	34 (51.5%)	32 (48.5%)	
PBI = 2	11 (35.5%)	20 (64.5%)	

CI = Caries Index, PBI = Papillary Bleeding Index.

**Table 2:** Individual relation of CI with oxidative stress markers, age and plaque index.

	CI = 0 Average ± Standard Deviation	CI = 2 Average ± Standard Deviation	*p*-Value
Age	4.4 ± 9.8	9.4 ± 4.5	0.39
Age (3–12)	7.6 ± 2.6	7.1 ± 2.4	0.34
Age (13–18)	16 ± 1.1	16.3 ± 1.2	0.33
Plaque index	24.3 ± 11.3	30.1 ± 16.5	0.04 *
Plaque index (3–12)	23.7 ± 11.4	27.4 ± 15.5	0.27
Plaque index (13–18)	25.5 ± 11.4	35.2 ± 17.5	0.05
TBARS (µmol/L)	3 ± 1.4	3.1 ± 1.4	0.75
TBARS (3–12) (µmol/L)	2.9 ± 1.4	3.2 ± 1.3	0.28
TBARS (13–18) (µmol/L)	3.4 ± 1.1	2.9 ± 1.5	0.21
AOPP (µmol/L)	75.9 ± 65.3	71.8 ± 78	0.48
AOPP (3–12) (µmol/L)	74.6 ± 67.6	74.6 ± 85.1	0.99
AOPP (13–18) (µmol/L)	79.8 ± 59.6	63.4 ± 53.3	0.36
TAC (µmol/L)	541.4 ± 228	522.5 ± 204.1	0.57
TAC (3–12) (µmol/L)	494.1 ± 229.7	520 ± 216	0.52
TAC (13–18) (µmol/L)	675 ± 163.9	530.1 ± 166.8	0.008 *

* Indicates *p*-value < 0.05.

**Table 3:** Individual PBI relation to oxidative stress markers, age and plaque index.

	PBI = 2 Average ± Standard Deviation	PBI = 1 Average ± Standard Deviation	PBI = 0 Average ± Standard Deviation	*p*-Value
Age	14.1 ± 2.9	10.8 ± 3.2	5.25 ± 1.9	0.0001 *
Plaque index	32.1 ± 16.7	25.4 ± 12.7	19.1 ± 7.8	0.03 *
TBARS (µmol/L)	3.1 ± 1.1	3.1 ± 1.5	3 ± 1.2	0.97
AOPP (µmol/L)	84.5 ± 90.9	68.4 ± 75.3	73.7 ± 54.8	0.31
TAC (µmol/L)	623.4 ± 176.3	507.3 ± 210.4	509.2 ± 233.2	0.02 *

* Indicates *p*-value < 0.05.

## Data Availability

The data used to support the findings are available from the corresponding authors upon reasonable request.
